# Growing climate change risk concerns with rising regional disparities in China

**DOI:** 10.1038/s44168-025-00272-z

**Published:** 2025-08-20

**Authors:** Ziqian Xia, Jinquan Ye, Ramit Debnath, Xu Dong, Jinliang Xie, Ming Xu, Xi Tian, Jennifer Marlon, Chao Zhang, Jianxun Yang, Sara Constantino, Miaomiao Liu

**Affiliations:** 1 School of Economics and Management, Tongji University, Shanghai, China; 2 Doerr School of Sustainability, Stanford University, Stanford, CA USA; 3 Collective Intelligence & Design Group, University of Cambridge, Cambridge, UK; 4 Nicholas School of the Environment, Duke University, Durham, NC USA; 5 Climate and Social Intelligence Lab, Caltech, Pasadena, CA USA; 6 Department of Geography, National University of Singapore, Singapore, Singapore; 7 School of Environment, Tsinghua University, Beijing, China; 8 School of Economics and Management, Nanchang University, Nanchang, China; 9 School of the Environment, Yale University, New Haven, CT USA; 10 State Key Laboratory of Water Pollution Control and Green Resource Recycling, School of the Environment, Nanjing University, Nanjing, China; 11 Institute for the Environment and Health, Nanjing University Suzhou Campus, Suzhou, China

**Keywords:** Environmental social sciences, Climate-change adaptation, Climate-change impacts, Climate-change policy, Psychology and behaviour

## Abstract

This study presents a high-resolution mapping of climate change perceptions across China, highlighting the evolution of public perception regarding the priority and impact of climate change over a 13-year period between 2010 and 2023. Utilizing data from two national surveys conducted (*N* = 11783 and *N* = 4050), we show a considerable rise in the perceived priority (19%) and impact (13%) of climate change issues nationally, alongside growing regional disparities. We do robustness checks of our results using repeated simulations between multilevel regression and poststratification and disaggregation methods. By examining perceived impacts against actual risk exposure, we show the need for managing regional vulnerabilities and tailored and targeted communication strategies to mitigate the spatial mismatch between climate change perception and risk exposure.

## Introduction

Climate change poses an escalating threat to China’s society and economy, yet public engagement with this issue has not kept pace with the rising risks^1^. China has been warming faster than the global average (about 1.3–1.6 °C rise over the past century, versus <1 °C globally)^2^. As a result, the country is experiencing more frequent heat waves and intense rainfall events, evidenced by recent record-breaking summer heat and catastrophic floods^3^. Prior literature suggests that exposure to extreme weather may shape concern about climate change, as well as behavioral and policy outcomes, though the evidence is mixed and the relationship is likely to depend on other moderating factors (e.g., media environment, responsiveness of local government)^4,5^. As a growing number of Chinese residents are exposed to these events, concern among Chinese residents may be growing. Yet much of the work to date on extreme weather exposure and risk perceptions has been focused on the Global North, and there has been very limited work on the beliefs and attitudes of Chinese publics—especially with regional granularity. As a result, we lack a high‑resolution picture of where concern is highest, where it lags behind objective hazard risks, and how these spatial patterns have evolved over time in the world’s largest greenhouse‑gas emitting country. Understanding these dynamics is important because China’s pathway to peak‑carbon‑before‑2030 and carbon‑neutrality‑by‑2060 will ultimately be executed by 31 provinces and more than 300 prefecture‑level cities, each facing distinct climate hazards, economic profiles, adaptive capacities, and public opinion. Whether local publics perceive climate change as an urgent risk can accelerate—or obstruct—provincial mitigation and adaptation plans—with implications for global decarbonization goals^6,7^.

Existing research suggests that climate change awareness in China has historically lagged behind developed countries, but has been rapidly growing in recent years. In 2008 only about 62% of Chinese adults had heard of climate change^8^, compared to over 90% in North America^9^, Europe and Japan^8^. Most of these surveys, however, were national polls or city case studies that obscure important subnational heterogeneity in public opinion. Additionally, the studies rarely pair opinion with objective climate-risk metrics^8,10^.

To close this evidence gap, we develop a multilevel regression and poststratification (MRP) model to understand: *How Chinese citizens perceive climate change? How these perceptions vary over time and across the country’s regions? Whether those perceptions align with objective heat-risk exposure?*

While exposure to extreme weather has been hypothesized to increase concern about climate change, the evidence is mixed and has been shown to depend on other psychological, social and contextual factors. Drawing on the concept of the risk society^11–13^, climate concern might depart from exposure as risk perceptions are socially mediated and institutionally embedded. In practice, provinces with high institutional trust in government and scientists are more likely to accept official climate warnings and endorse mitigation policies^14,15^. Greater scientific literacy—often proxied by tertiary-education rates—enhances people’s ability to process complex climate information and recognize long-term risks^16–18^. Finally, a robust media environment can amplify risk signals by attributing extreme events to climate change, whereas information-poor settings may leave hazards unexplained and concern muted^19,20^. Guided by this framework, we test two interlinked hypotheses to unravel the spatial dynamics of climate concern in China. Note that we do not test these mechanisms formally in this study; rather, we invoke them to explain why climate perceptions might diverge across China’s regions. They provide the theoretical lens through which we interpret the high-resolution maps that follow, helping to make sense of the sharp provincial differences our descriptive analysis reveals. Thus, our H1 posits that public awareness of climate change varies systematically across China’s regions—even after accounting for the nationwide rise in awareness from 2010 to 2023. H2 posits that provincial rankings of perceived climate-change impact will diverge from rankings of objective heat-risk exposure.

We created a data-driven and high-resolution map^21^ of climate change perception in the country at both provincial and prefecture-city levels based on two national surveys conducted in 2010 and 2023 (*N* = 11783 and *N* = 4050, respectively). By juxtaposing opinion maps with climate risk maps, we can pinpoint “perception gaps”—areas where climate change is having large impacts but public concern remains relatively low, and vice versa. Identifying these gaps can provide insights about where to target climate communication and policy interventions.

## Results

### Nationwide rise in climate change concern

**Fig. 1:**
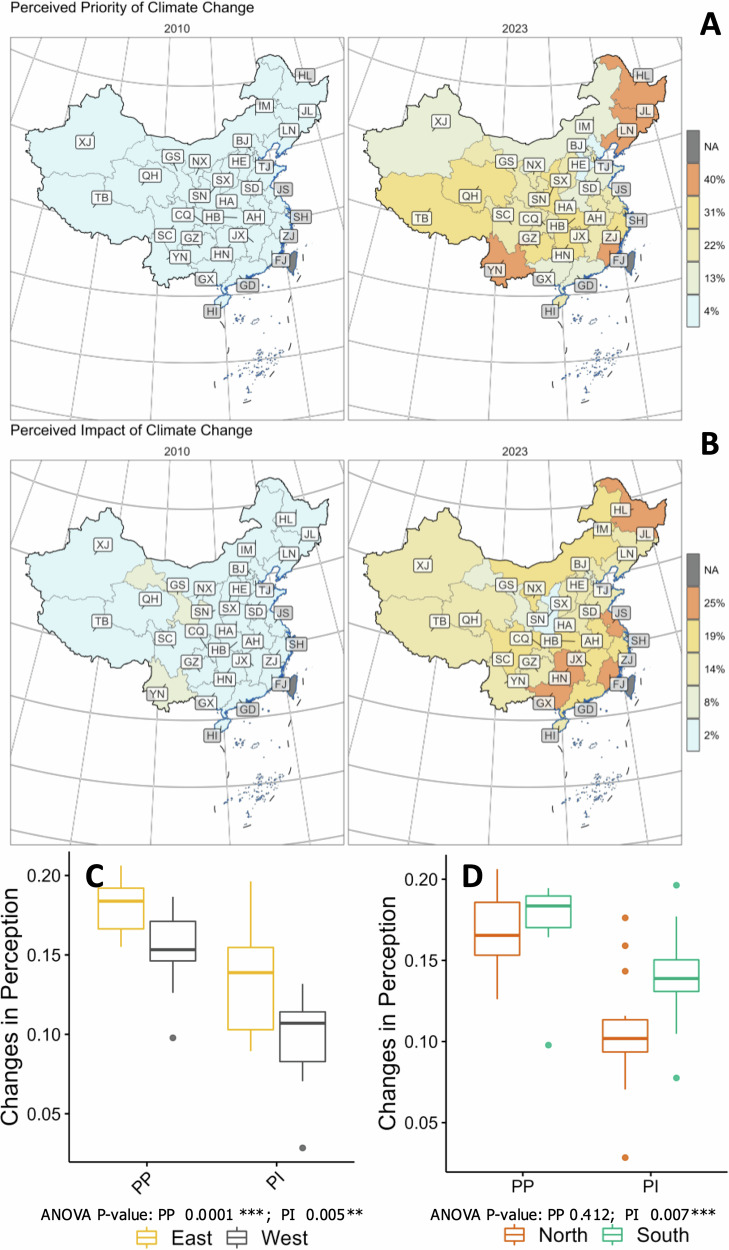
Climate change perceptions in China evolved differently across regions from 2010 to 2023. Provincial estimates of percentages of individuals who perceive climate change as **A** the most urgent environmental problem (perceived priority) and **B** having the most severe consequences (perceived impact) in 2010 and 2023, respectively. Boxplots compare the scale of temporal shifts in perceived priority and impact of climate change from 2010 to 2023 across **C** the Eastern and Western regions, and **D** the Northern and Southern regions in China.

We deployed the MRP model to generate reliable estimates of climate change perceptions at both provincial and prefecture-city levels^22^. We observed that the perceived priority (PP) of climate change has risen in all provinces across China (see Fig. 1A), as revealed by our analysis using the location-scale model^23^. Over the 13-year span from 2010 to 2023, public concern about climate change in China grew markedly. In 2010, climate change barely registered as a top environmental priority for most Chinese—on average only around one in twenty respondents considered it the nation’s most urgent environmental problem (5.91% in 2010). By 2023, that share had surged to nearly one in four (23.4% in 2023; *t* = 19.84, *P* < 0.001; disaggregation methods). Specifically, in 2010, the percentage of residents viewing climate change as the top environmental issue ranged from 4.4% in Beijing (BJ), China’s capital, to 11.5% in Yunnan (YN), one of the least developed provinces in southwestern China. In 2023, this range increased to 16.9% in Guangdong (GD), a more developed southern coastal province with the largest population, to 40.1% in Liaoning (LN), the most populous province in the northeastern coastal region.

A similar jump occurred in perceived personal impact: in 2010 only ~4% of the public believed climate change would have the greatest impact on their family compared to other environmental issues, whereas by 2023 this had quadrupled to about 16% (*t* = 25.45, *P* < 0.001). Such increases reflect a broad-based rise in climate awareness across the country. In 2010, people who believed that climate change would have the greatest impacts on individual and its family ranged from 2.4% in Beijing (BJ) to 8.4% in Yunnan (YN). By 2023, these percentages increased to a range of 12.9% in Shandong (SD), a province renowned for its significant coastal climate influence in East China, to 22.9% in Heilongjiang (HL), a province characterized by having the lowest average temperatures nationally and frequent extreme cold weather events. This observed uptick in climate change perception is likely influenced by factors such as enhanced media coverage^24^, increased personal experiences with climate events^25^, and initial levels of climate awareness.

### Regional disparities in growth of Chinese climate change perceptions

Our high-resolution maps reveal that China’s rising aggregate climate concern masks substantial geographic heterogeneity. In support of H1, this trend is underscored by the rise in the logarithmic standard deviation of provincial perception percentages, which serve as a measure of variability. Specifically, the deviation for perceived priority (PP) increased by 0.999 (*t* = 5.437, *p* < 0.001), and for perceived impact (PI), it rose by 0.977 (*t* = 5.563, *p* < 0.001), highlighting growing regional differences in climate change perceptions. Analysis of Variance (ANOVA) tests were used to compare the 13-year changes in PP and PI between two pairs of province groups (i.e., Northern and Southern provinces, Eastern and Western provinces) to investigate how the estimated growth in climate change perception differed across regions (see Fig. 1C, D). Regions were divided according to conventions commonly adopted in previous studies^26,27^. Our results show that survey respondents living in Eastern China experienced more pronounced increases in PP (*F* = 17.090, *p* < 0.001) and PI (*F* = 8.797, *p* < 0.01) compared to those in the West. Views of respondents in the Southern China have grown faster for PI (*F* = 8.341, *P* < 0.01), but not for PP (*F* = 0.693, *P* = 0.412).

**Fig. 2:**
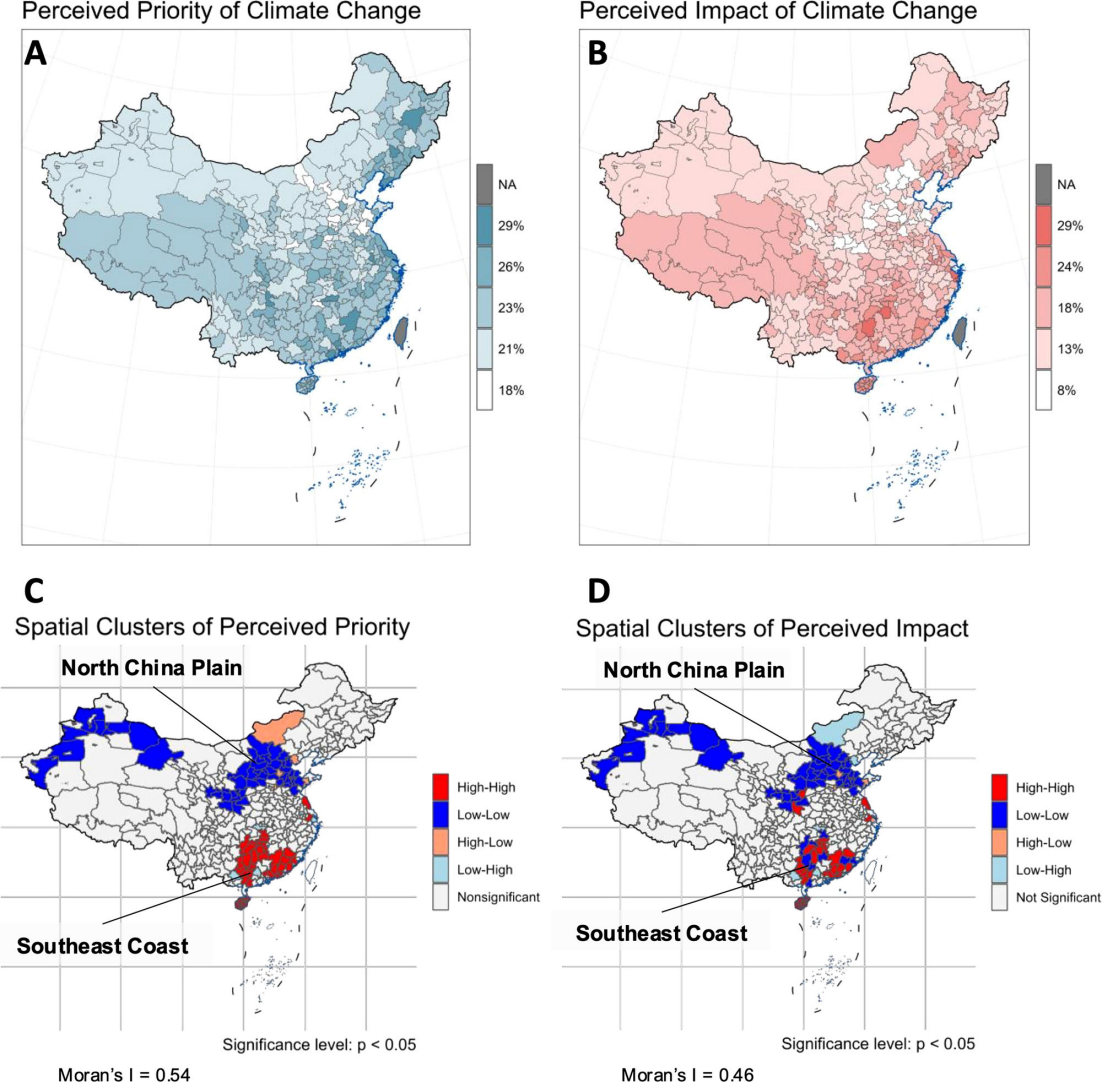
City-level estimates of climate change perception and spatial clusters. **A** Percentage of the population who perceive climate change as the priority (PP) at the city level. **B** Percentage of population who think climate change will lead to the most severe impacts (PI) at the city level. Spatial cluster maps (**C**) and (**D**) show hot spots and cold spots for PP and PI, respectively. Red regions denote significant city clusters with high PP and PI (high-high clusters). Blue regions denote city clusters with low PP and PI (low-low clusters).

This disparity in climate change perceptions across China is further corroborated by our detailed city-level perception map and spatial analysis for 2023 (see Fig. 2A, C). Our results reveal significant heterogeneity among cities, with the percentage of the population identifying climate change as a priority ranging from a low of 18% to a high of 28% at the city level, indicating a 10% gap. The perception of climate change impact shows even greater variations, ranging from 7% to 29% across cities, a substantial 22% gap. Spatial autocorrelation analysis revealed distinct patterns: significant clusters where cities either consistently reported high (“High-High”) or low (“Low-Low”) levels of climate change perception. These high-perception clusters predominantly appeared along China’s southern and eastern coasts, while lower-perception clusters were found mainly in the northern and central plains and in western Xinjiang (Fig.2B, D).

### Spatial mismatch between impact perception and actual climate risk

**Fig. 3:**
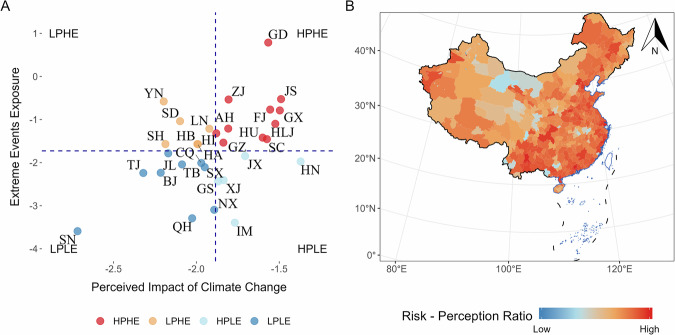
Provincial and city-level analysis of perceived impact and exposure to extreme events. **A** The provincial map visualizes the relationship between perceived impact of climate change and exposure to extreme weather events, illustrating the spatial distribution of climate risk across China. Here, HPHE (High Perception-High Exposure) regions are those where residents experience high levels of actual climate risk and also have a high level of perception regarding climate impacts. Conversely, LPHE (Low Perception-High Exposure) regions are areas where residents hold low perceptions of climate change impacts despite facing high levels of actual climate risk. The extreme weather events are defined based on the 98th percentile of historical daily mean temperatures for each province from 1980 to 2020. **B** The city-level map represents a finer scale down of the risk-perception ratio, highlighting the discrepancies between perceived risk and actual exposure to extreme weather events. The risk-perception ratio is computed by dividing the perceived impact of climate change on families by the exposure to extreme events.

Perhaps most critically, our results uncover mismatches between objective climate risks and public risk perceptions across China’s provinces. We juxtaposed each province’s climate hazard exposure—measured here by the frequency of extreme temperature anomalies—with its residents’ PI of climate change. Note that “exposure” denotes the level of population vulnerability, and “PI” indicate the public’s perceived effects of climate change on individuals and families. We interpret the results of our vulnerability assessment based on the PI of climate change^28^, as well as population exposure to extreme events in China. We classify the provinces into four categories (see Fig. 3A and B) according to their PI and actual exposure to extreme events (exposure). Monitoring changes in exposure and ensuring residents are informed and prepared for potential future risks should be prioritized. Such spatial mismatches highlight that higher climate risk does not automatically translate into higher perceived risk. Understanding these gaps is vital: regions where climate threats are underestimated by the public could face greater vulnerability if complacency delays adaptation measures. Likewise, areas with high perceived risk but comparatively lower exposure might reflect successful risk communication or broader environmental awareness that policymakers can learn from.

### Robustness checks and model validation

**Fig. 4:**
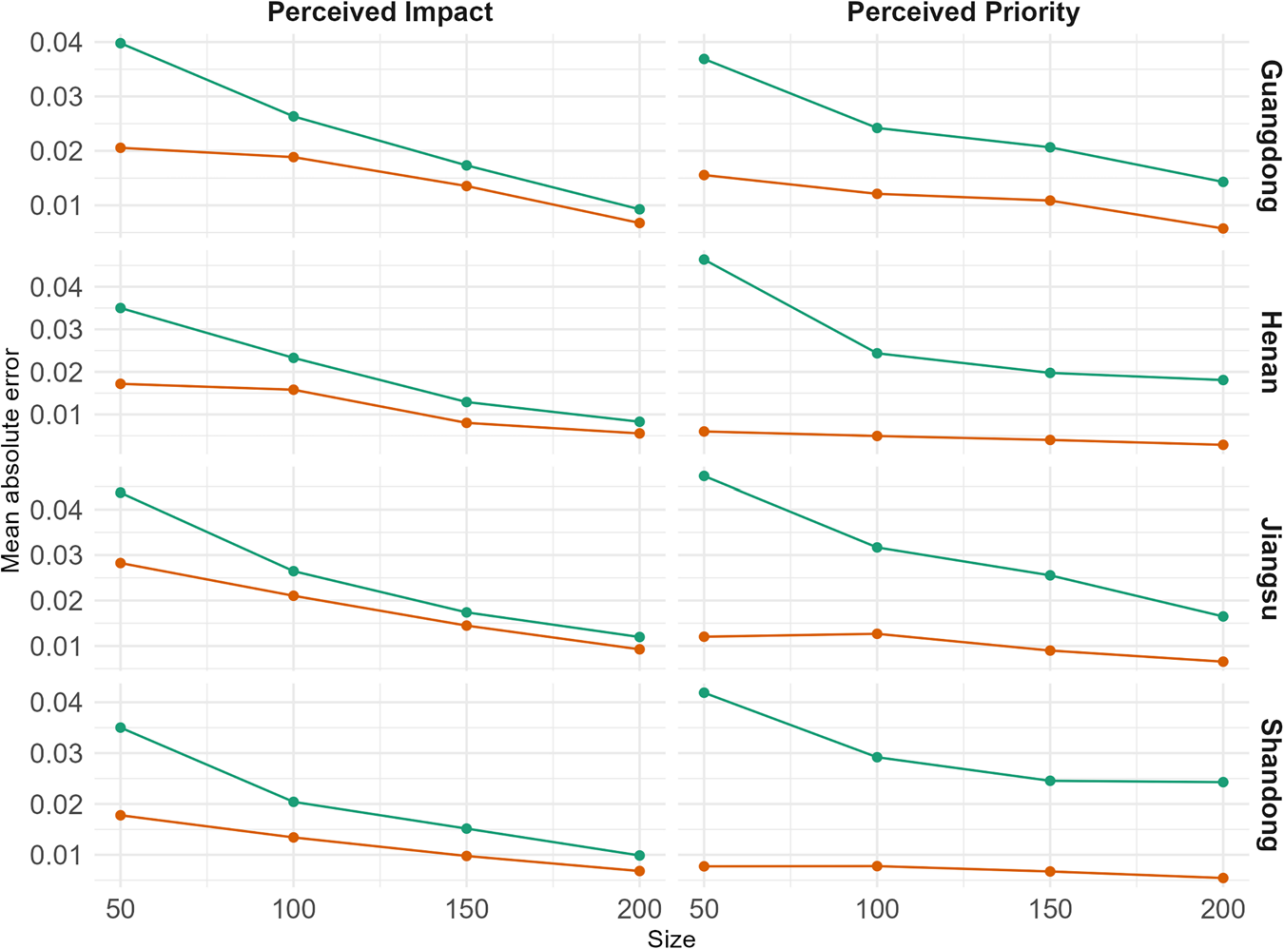
Mean absolute error (MAE) comparison between multilevel regression and poststratification (MRP) and disaggregation methods over repeated simulations. Y-axis is the MAE of the model; X-axis is the index of 99 random samplings of 50, 100, 150, and 200.

To ensure the reliability and validity of our findings, we conducted several robustness checks and model validations, following the method of previous studies^29^. First, we compared the estimates derived from MRP against those obtained through a Disaggregation method. Figure 4 illustrates the Mean Absolute Error (MAE) for PP and PI of climate change, derived from both methods across various sample sizes and provinces. The sample sizes were determined based on 99 random samplings of 50, 100, 150, and 200 responses from the survey data. The results indicate that MRP consistently outperformed the Disaggregation method in terms of MAE, demonstrating its robustness across different sample sizes. In this case, MRP is effective in accurately estimating climate change perceptions, even when working with smaller sample sizes.

## Discussion

Why, then, do provinces display such different levels of concern? Risk-perception studies point to four amplifiers—institutional trust, scientific literacy, media exposure, and economic development—and China’s provincial mosaic offers vivid examples^8,24^. Guangdong—a coastal manufacturing powerhouse of 127 million residents with some of the country’s highest university-enrollment and media-penetration rates—shows the steepest rise in PP; here typhoon coverage and science-based messaging appear to translate exposure into heightened concern. By contrast, Henan, an agrarian province on the central plains with lower tertiary-education rates, exhibits only modest gains despite similar or greater heat stress, suggesting that livelihoods and informational capacity moderate risk signals^10,24^. When comparing with actual risk, Hubei (central China’s riverine logistics hub) sits in the low-perception/high-exposure quadrant, a likely blind spot tied to weaker climate framing in local media; Inner Mongolia (northern rangelands) illustrates high-perception/low-exposure, where pastoral livelihoods make residents acutely sensitive to even small climatic shifts^30^. Such decouplings are not unique to China. In the United States, surveys find only a weak correlation between people’s perceived threat and their actual local climate hazards and strong misalignment^31,32^. Across Europe, countries with comparable climate risks exhibit divergent concern levels—notably, public concern in Eastern Europe remains markedly lower than in Western Europe despite growing climate impacts^33^. Globally, many highly vulnerable developing regions (the Global South) still report low climate-change awareness, underscoring an awareness gap between high-risk areas and public understanding^34,35^. Together, these patterns confirm that social and informational contexts, not hazard exposure alone, shape the geography of climate concern.

Our analysis also reveals a complex and dynamic interaction between perceived climate risk and real climate hazard in China over time (Supplementary Fig. S4). Again, the Social Amplification of Risk Framework provides a coherent theoretical lens^36,37^: when heat-waves or typhoons are explicitly linked to climate change by local newsrooms, social-media influencers, and trusted officials, the hazard signal is amplified and public concern rises toward the objective risk; when the same events are framed as routine weather or receive little coverage, the signal is attenuated and the mismatch persists. SARF thus clarifies why perception–risk mismatches cluster around “amplification” or “attenuation” stations such as regional newsrooms, social‑media feeds, schools, and leadership forums, rather than mirroring physical exposure alone^24^.

It is important to recognize that raising public risk perception, while essential, is not a panacea for engendering climate action. Our results show a significant increase in the Chinese public’s prioritization and PI of climate change—an encouraging trend that could increase support for pro-climate behaviors and policies. However, behavioral theory and past studies caution that perception alone has limits in spurring action^38–40^. Even when people acknowledge risks, there are often psychological and practical barriers that prevent them from changing behavior or supporting difficult policy measures. This widely documented attitude–behavior gap means that concern does not automatically translate into actions^41^. In China, and in many developing countries, one must consider that despite more citizens recognizing climate change as urgent, behavioral plasticity may be limited and economic and livelihood concerns may be prioritized over climate change^42,43^. For example, a farmer in a drought-prone northern province might agree that climate change poses a serious threat, yet feel unable to adopt adaptive practices due to cost or lack of knowledge—or simply have more immediate daily concerns. Likewise, an urban resident might be worried about climate change but not reduce personal car use without viable alternatives. Climate change is a “manufactured” risk that citizens expect expert systems to manage^13^; rising awareness can therefore coexist with a reliance on institutional solutions rather than personal change. Converting concern into action requires additional enabling factors—accessible options, economic incentives, supportive policies, and continual engagement—beyond just awareness.

Our study also brings to the fore that successful climate change communication in China must be localized. Communication campaigns can utilize local narratives, indigenous language, and case studies from the community to make the connection real^44–46^. Localized policy interventions need to be embedded within the prevailing community structures to enable deeper and broader engagement^46,47^. Precision engagement like these is key to overcoming the perception–risk gap revealed through our research. Our maps at high resolution are employed as a diagnostic, enabling policymakers to identify places where there is immense public mismatch with climate risk, and track the impact of targeted interventions over time.

Our study has certain limitations. First, due to the unavailability of updated data from the Chinese General Social Survey (CGSS) for 2023, we relied on our own data collection. Although this allowed us to capture recent perceptions, it introduced potential discrepancies between our survey methodologies and those used in previous studies. To address this and ensure comparability, we employed MRP, a method known for producing reliable estimates from even biased or small sample sizes. However, while MRP is a robust tool, it cannot completely eliminate the possibility of biases introduced by differences in sample characteristics between the 2010 and 2023 datasets. Second, while our 2023 survey achieved a sample size of ~4000 respondents, there is still a possibility that the regional and demographic variations present in the sample could affect the generalizability of the findings. Although MRP helped mitigate these concerns, we acknowledge that this method, like any statistical model, is contingent on the quality and representativeness of the data fed into it. Moreover, given the complexity of climate change impacts, future research should broaden the scope to encompass multiple climate variables, such as extreme precipitation and storm events, alongside temperature increases. This would provide a more comprehensive view of how different facets of climate change are perceived and their respective influence on risk awareness. Lastly, while our research highlights significant shifts in climate change perceptions, we recognize that perception alone is not sufficient to drive behavioral change or prompt widespread action. Perceptions of climate change urgency or impact do not necessarily translate into concrete mitigation or adaptation efforts. Future research should explore the gap between perception and action, as well as the underlying barriers preventing individuals from transitioning their awareness into effective responses to climate change.

To conclude, this research delivers the first high-resolution map of how climate change perceptions have evolved across China’s diverse regions, revealing a heartening increase in public sense of urgency about climate change. We find that climate change perception in China has grown from a fledgling concern into a prevalent issue for many citizens, mirroring the country’s intensified experience of climate impacts and its ramped-up climate policy efforts. At the same time, we illuminate widening regional gaps—a reminder that national level measurement encompasses many different climate-related risk realities and information landscapes. Our paper highlights this inner national disparity: Regions where climate concern remains low despite high risks must not be overlooked; otherwise, unequal awareness could exacerbate the unequal burden of climate impacts. Our hope is that these findings inform targeted strategies to raise climate change perception where it is needed most, thereby strengthening the social foundations for ambitious climate action.

## Methods

This study is framed around two primary objectives organized into two hypotheses. To address the first objective, we employed MRP to map the status and changes in climate change perception across China between 2010 and 2023, hypothesizing that significant growth in climate change awareness has occurred with substantial regional variability (H1). For the second objective, we conducted a descriptive comparison between actual climate risks and PI, hypothesizing that regions with higher exposure to climate risks do not necessarily exhibit stronger perceptions of climate change impact (H2). This methodological framework, together with data used, are introduced in the following sub-sections.

### Data

Two key questions were used to measure climate change perception. The first, “Is climate change the most urgent environmental problem in China?” (perceived priority) assessed the PP of climate change relative to other environmental issues. The second question, “Will climate change have the greatest impact on you and your families compared to other environmental issues?” (perceived impact) measured the perceived risk or impact (PI) of climate change. Two distinct datasets were used to provide estimate of climate perception, CGSS 2010 (*N* = 11,783) and our independent survey in 2023 (*N* = 4050).

CGSS is an annual, nationally representative survey that explores a wide range of aspects concerning social life in China. While the CGSS has been a crucial source of longitudinal data, it did not consistently include the specific questions related to climate change perception in its surveys post-2010. The CGSS’s 2021–2022 surveys, while valuable, lacked the particular items of interest to our study. Thus, to enable a direct comparison with the 2010 data using consistent measures, we found it necessary to undertake our own survey initiative. We retained the questions from the CGSS 2010 to ensure comparability. We carried out an independent online survey in January 2023, in partnership with WJX.cn, a Chinese online survey platform. To ensure the collection of high-quality data reflective of current public opinion on climate change, we established stringent quality control measures. These included the deployment of attention check questions to filter out non-serious responses and a detailed review process, where each submission was rigorously evaluated by two members of our research team for consistency and authenticity. We have documented ~4050 responses, striving for a diverse representation from across China’s regions. The sampling strategy, including the mode of survey, sample size, and demographic characteristics, are transparently compared in Table S1 of the Supplementary Information.

We also relied on a diverse set of data sources to ensure robustness and precision in our poststratification. For the provincial level analysis, data were sourced from China’s Seventh National Census (http://www.stats.gov.cn/). At the city level, data were meticulously drawn from various resources. Age and gender subgroup counts, critical components in our investigation, were obtained from a high-resolution (100 m) global grid-level age/sex population structure projection dataset for the year 2020, made available by the WorldPop Hub (https://hub-worldpop.opendata.arcgis.com/). Education subgroup population counts, essential for understanding the role of educational attainment in shaping climate change perceptions, were gleaned from the 2020 China Population and Employment Statistical Yearbook.

To complement our analysis of climate change perceptions with objective climate data, we obtained temperature records from the Global Summary of the Day (GSOD) dataset (https://www.ncei.noaa.gov/data/global-summary-of-the-day/access). The GSOD dataset provides daily meteorological observations from weather stations worldwide, including measurements of average, maximum, and minimum temperatures. For our study, we focused on temperature data from weather stations located within China. To derive city-level temperature estimates, we first interpolated the station-based temperature data using the Inverse Distance Weighting method^48^, generating gridded temperature data at a spatial resolution of 0.1˚ × 0.1˚. This interpolation method accounts for the spatial distribution of weather stations, ensuring that temperature estimates are representative of the broader geographic area. Subsequently, we aggregated the gridded temperature data to the city level by calculating the area-weighted average for each administrative boundary. This approach allowed us to obtain annual average, maximum, and minimum temperature values for each city in China, which were then used to analyze the relationship between local climate conditions and public perceptions of climate change.

### Multilevel regression and poststratification (MRP)

To estimate climate change perception in China, we used MRP^49,50^. This method is a form of survey weighting that allows researchers to make inferences about population-level characteristics based on a sample of respondents. It is particularly useful when studying subgroups or populations that are not well represented in the sample, as it allows researchers to adjust for any biases in the sample and make more accurate estimates^51,52^. MRP is even able to make relatively accurate estimate from highly selected biased samples^22,52,53^. Moreover, MRP can produce estimates using fairly simple demographic-geographic models of survey response and small amounts of survey data—as little as a single national poll^22,54^. MRP involves integrating both individual-level and group-level predictors into a statistical model fitted to the survey data. In our research, individual-level predictors comprised factors such as age, education, gender, and Hukou (residential status). Simultaneously, group-level predictors included regional GDP, historical extreme events, and local temperature. First, economic development is a well-established correlate of environmental and climate concern: cross-national analyses show that affluence and urbanization consistently raise the odds that citizens are aware of and worried about climate change^55^. GDP therefore captures a bundle of factors—education, media penetration, institutional capacity—that plausibly elevate climate salience. Second, repeated exposure to short-term extreme weather can act as an “experiential cue” that increases perceived climate risk, although the magnitude of this effect varies with local framing^44,56^. Third, local long-term temperature change is a parsimonious proxy for the physical signal of climate change that residents may observe^57,58^. The incorporation of these predictors allowed us to accommodate differences between individuals and regions, thereby enhancing the precision of our estimates of climate change perception across China. Empirically MRP is framed as :

1 $${\rm{Pr}} \left({Y}_{i}=1\right)={{\rm{logit}}}^{-1}\left(\begin{array}{c}{\gamma }_{0}+{\alpha }_{j\left[{i}\right]}^{{age}}+{\alpha }_{k\left[{i}\right]}^{{education}}\\ +{\alpha }_{\left[\right.[i]}^{{gender}}+{\alpha }_{{m}\left[{i}\right]}^{{Hukou}}+{\alpha }_{{j}\left[{i}\right],{k}\left[{i}\right],\left[{ii}\right]}^{{age.education.gender.Hukou}}+{\alpha }_{{c}\left[{i}\right]}^{{county}}\end{array}\right)$$

where

$$\begin{array}{c}{\alpha }_{j}^{\text{age}}\sim N\left(0,{\sigma }_{\text{age}}^{2}\right),{\text{for}}\,j=1,\ldots ,3\\ {\alpha }_{k}^{\text{education}}\sim N\left(0,{\sigma }_{\text{education}}^{2}\right),{\text{for}}\,k=1,\ldots ,8\\ {\alpha }_{l}^{\text{gender}}\sim N\left(0,{\sigma }_{\text{gender}}^{2}\right),{\text{for}}\,l={1,2}\\ {\alpha }_{m}^{\text{Hukou}}\sim N\left(0,{\sigma }_{\text{Hukou}}^{2}\right),{\text{for}}\,m={1,2}\\ {\alpha }_{j,k,l,r}^{\text{age.education.gender.Hukou}}\sim N\left(0,{\sigma }_{\text{age.education.gender.Houku}}^{2}\right),\\ {\text{for}}\,j=1,\ldots ,3{;k}=1,\ldots ,8{;l}={1,2}{;r}={1,2}\end{array}$$

Where $Y$ is the outcome variable (in this case, climate change perception), demographic factors such as age, gender, and educational attainment, as well as the interaction terms of these variables are considered as random effects. Geographical grouping is also considered by defining individuals based on their province and city to evaluate the random intercepts.

One of the key advantages of MRP is that it allows researchers to make estimates for subgroups or populations that are not well represented in the sample, by using information from external data sources to adjust the weights of the respondents. This is known as “poststratification,” and it allows researchers to make more accurate estimates of population-level characteristics, even when the sample is not representative of the population^22^. For internal cross-validation of our MRP estimates, we adhered to procedures established in prior research^9,32,59^, comparing our results to estimates derived from disaggregation using repeated simulations in which subsamples of varying sizes were randomly selected from larger-population provinces (see Fig. 4).

After applying the MRP technique, we performed spatial data exploration using the Local Indicators of Spatial Association (LISA) and Moran’s I. Moran’s I, a global measure of spatial autocorrelation, provides an indication of the degree of similarity between a given spatial unit and its neighbors across the entire study area^60,61^. It ranges from −1 (indicating perfect dispersion) to +1 (indicating perfect correlation). A zero value suggests a random spatial pattern. We applied the global Moran’s I statistic to detect overall patterns of spatial clustering of climate change perceptions at the city level in China.

To supplement the global view provided by Moran’s I, we employed LISA statistics, which provide a local perspective of spatial autocorrelation^62^. LISA identifies regions where neighboring areas show similar values (high-high or low-low), termed positive spatial autocorrelation, or dissimilar values (high-low or low-high), termed negative spatial autocorrelation.

### Location scale model

To examine the temporal variation in PI and PP of climate change, we employed a Location Scale Model (LSM). The LSM is a statistical tool that facilitates the assessment of not only the central tendency (or location) of a given distribution but also the scale (or variability) within that distribution over time^23,63,64^. This allows the LSM to reveal changes in both the mean values and the heterogeneity of public perceptions.

In the context of our study, the LSM helped decipher the changing patterns of climate change perceptions across China over the studied period. The LSM was particularly useful in quantifying and comparing the heterogeneity within these perceptions at different points in time (2010 vs. 2023), providing an additional layer of understanding beyond the mere average changes. This enhanced our capacity to capture the intricate dynamics of public sentiment towards climate change. In statistical terms, the LSM can be represented as follows:

2 $$Y=\mu +\sigma \varepsilon$$

where $Y$ is the outcome variable (PP or PI), $\mu$ is the location parameter that represents the population mean of the outcome variable, $\sigma$ is the scale parameter that signifies the standard deviation of the outcome variable, and ε is the error term following a standard normal distribution. By analyzing both parameters over time, we could discern whether public climate change perceptions in China were not only shifting on average but also becoming more or less disperse.

### Exposure and risk-perception ratio calculation

In accordance with the Intergovernmental Panel on Climate Change Sixth Assessment Report (IPCC AR6) risk framework^65^, we define exposure as the presence of individuals and communities in areas where they are subject to climate hazards, while vulnerability refers to their susceptibility to adverse effects due to social, economic, and environmental factors. In our study, exposure is calculated independently from vulnerability, focusing on heat stress as a key climate risk indicator. As noted in IPCC AR6, heat stress^66^—measured as the number of people exposed to extreme heat events—is the main focus of our analysis.

To quantify exposure, we first calculated the climate risk for specific cities using exposure rates to extreme temperature events. The threshold for extreme events was determined based on the 98th percentile of the historic daily mean temperature in each city from 1980 to 2020. For instance, if the 98th percentile of daily mean temperature is 35 °C, any day with a mean temperature above 35 °C would be considered an extreme event. The extreme event threshold for each city is calculated as follows:

3 $${T}_{{extreme}}={P}_{98}\left({T}_{{mean}}\right)$$

Where ${T}_{{extreme}}$ is the extreme event threshold, ${P}_{98}$ is the 98th percentile, and ${T}_{{mean}}$ is the daily mean temperature. Next, we used population data to calculate the exposure to extreme events in each city. The exposure was calculated by multiplying the number of extreme event days by the population of the city. The exposure formula is as follows:

4 $$E={N}_{{extreme}}\times P$$

Where $E$ is the exposure, ${N}_{{extreme}}$ is the number of extreme event days, and $P$ is the city’s population. We then calculated the risk-perception ratio (PRR) by dividing the perception of climate change’s impact on families by the exposure to extreme events. The risk-perception ratio was mapped to visualize the need for action in each region. It is calculated as follows:

5 $${RPR}=\frac{{PI}}{E}$$

Where ${PI}$ is PI, $E$ is the exposure.

## Supplementary information

Supplementary materials-SUBMIT

## Data Availability

The primary data for this study are derived from the Chinese General Social Survey (CGSS) 2010 and a separate online survey conducted by our research team in 2023. Due to privacy restrictions and data agreements, these datasets are not publicly available. However, interested researchers can obtain CGSS 2010 data access through the formal application to the Chinese Social Survey Data Center, Renmin University of China. The 2023 survey data can be requested directly from the corresponding author, subject to ethical considerations and data usage agreements. The external datasets utilized for poststratification, including China's Seventh National Census and WorldPop Hub's high-resolution (100m) global grid-level age/sex population structure projection dataset for 2020, are publicly available through their respective sources. The temperature data used in this study, sourced from the Global Summary of the Day (GSOD) dataset. Any additional derived data supporting the findings of this study and the custom codes written for the analysis are available from the corresponding author upon reasonable request.

## Code availability

The custom codes written for the analysis in this study are available from the corresponding author upon reasonable request.

## References

[1] Pollard, M. Q. & Pollard, M. Q. China avoids climate change discussion despite extreme weather. *Reuters* (2023).

[2] Zhao, W. Extreme weather and climate events in China under changing climate. *Natl. Sci. Rev.* **7**, 938–943 (2020). 34692114 10.1093/nsr/nwaa069 PMC8291369

[3] Coumou, D. & Rahmstorf, S. A decade of weather extremes. *Nat. Clim. Chang.* **2**, 491–496 (2012).

[4] Howe, P. D. Extreme weather experience and climate change opinion. *Curr. Opin. Behav. Sci.* **42**, 127–131 (2021).

[5] Egan, P. J. & Mullin, M. Turning personal experience into political attitudes: the effect of local weather on Americans’ perceptions about global warming. *J. Polit.* **74**, 796–809 (2012).

[6] Gazmararian, A. F., Mildenberger M. & Tingley, D. Public opinion foundations of the clean energy transition. *Environ. Polit*. **0**, 1–23.

[7] Shen, C. & Wang, Y. How does public concern about climate change affect carbon emissions? Evidence from large-scale online content and provincial-level data in China. *J. Clean. Prod.* **426**, 139137 (2023).

[8] Yang, J., Gounaridis, D., Liu, M., Bi, J. & Newell, J. P. Perceptions of climate change in China: evidence from surveys of residents in six cities. *Earths Future* **9**, e2021EF002144 (2021).

[9] Howe, P. D., Mildenberger, M., Marlon, J. R. & Leiserowitz, A. Geographic variation in opinions on climate change at state and local scales in the USA. *Nat. Clim. Chang.* **5**, 596–603 (2015).

[10] Wang, B. & Zhou, Q. Climate change in the Chinese mind: an overview of public perceptions at macro and micro levels. *WIREs Clim. Chang* **11**, e639 (2020).

[11] Beck, U. & Sznaider, N. Unpacking cosmopolitanism for the social sciences: a research agenda. *Br. J. Sociol.* **57**, 1–23 (2006). 16506994 10.1111/j.1468-4446.2006.00091.x

[12] Giddens, A. Risk and Responsibility. *Mod. Law Rev.* **62**, 1–10 (1999).

[13] Beck, U. & Beck, U. *Risk Society: Towards a New Modernity* (Sage, 2009).

[14] Cologna, V. & Siegrist, M. The role of trust for climate change mitigation and adaptation behaviour: a meta-analysis. *J. Environ. Psychol.* **69**, 101428 (2020).

[15] Pan, Y., Xie, Y., Jia, H., Luo, X. & Zhang, R. Lower carbon, stronger nation: exploring sociopolitical determinants for the Chinese public’s climate attitudes. *Int. J. Environ. Res. Public. Health* **20**, 57 (2022). 36612381 10.3390/ijerph20010057 PMC9819301

[16] Pan, W.-L. et al. The role of climate literacy in individual response to climate change: evidence from China. *J. Clean. Prod.* **405**, 136874 (2023).

[17] Hu, C., Pan, W., Wen, L. & Pan, W. Can climate literacy decrease the gap between pro-environmental intention and behaviour? *J. Environ. Manage.* **373**, 123929 (2025). 39740442 10.1016/j.jenvman.2024.123929

[18] Kahan, D. M. et al. The polarizing impact of science literacy and numeracy on perceived climate change risks. *Nat. Clim. Chang.* **2**, 732–735 (2012).

[19] Lidskog, R., Soneryd, L. & Uggla, Y. *Transboundary Risk Governance*. 10.4324/9781849774642 (Routledge, 2009).

[20] Adger, W. N., Barnett, J., Brown, K., Marshall, N. & O’Brien, K. Cultural dimensions of climate change impacts and adaptation. *Nat. Clim. Chang.* **3**, 112–117 (2013).

[21] Park, D. K., Gelman, A. & Bafumi, J. Bayesian multilevel estimation with poststratification: State-level estimates from national polls. *Polit. Anal.* **12**, 375–385 (2004).

[22] Lax, Jeffrey, R., & Justin, H. Phillips. How Should We Estimate Sub-National Opinion Using MRP. *Preliminary Findings and Recommendations* 39 (2013).

[23] Rigby, R. A. & Stasinopoulos, D. M. Generalized additive models for location, scale and shape. *J. R. Stat. Soc. Ser. C Appl. Stat.* **54**, 507–554 (2005).

[24] Wang, X. Understanding climate change risk perceptions in China: media use, personal experience, and cultural worldviews. *Sci. Commun.* **39**, 291–312 (2017).

[25] Xia, Z. et al. A meta-analysis of the relationship between climate change experience and climate change perception. *Environ. Res. Commun.* **4**, 105005 (2022).

[26] Zhang, L., Sun, P., Huettmann, F. & Liu, S. Where should China practice forestry in a warming world? *Glob. Chang. Biol.* **28**, 2461–2475 (2022). 34962005 10.1111/gcb.16065

[27] Ge, J. & Xie, Z. Geographical and climatic gradients of evergreen versus deciduous broad-leaved tree species in subtropical China: implications for the definition of the mixed forest. *Ecol. Evol.* **7**, 3636–3644 (2017). 28616161 10.1002/ece3.2967 PMC5468137

[28] Huang, J., Yu, H., Guan, X., Wang, G. & Guo, R. Accelerated dryland expansion under climate change. *Nat. Clim. Chang.* **6**, 166–171 (2016).

[29] Mildenberger, M. et al. The distribution of climate change public opinion in Canada. *PLoS ONE* **11**, e0159774 (2016). 27486659 10.1371/journal.pone.0159774 PMC4972305

[30] Sattler, D. N., Bishkhorloo, B. & Graham, J. M. Climate change threatens nomadic herding in Mongolia: a model of climate change risk perception and behavioral adaptation. *J. Environ. Psychol.* **75**, 101620 (2021).

[31] Hunt, L., Williamson, M. & Hillis, V. Spatial (mis)alignment between climate-related risks and risk perceptions across the US. *Environ. Res. Lett.* **18**, 021001 (2023).

[32] Marlon, J. R. et al. Change in US state-level public opinion about climate change: 2008–2020. *Environ. Res. Lett.* **17**, 124046 (2022).

[33] Ergun, S. J., Karadeniz, Z. D. & Rivas, M. F. Climate change risk perception in Europe: country-level factors and gender differences. *Humanit. Soc. Sci. Commun.* **11**, 1–13 (2024).

[34] Spektor, M., Fasolin, G. N. & Camargo, J. Climate change beliefs and their correlates in Latin America. *Nat. Commun.* **14**, 7241 (2023). 37945560 10.1038/s41467-023-42729-x PMC10636181

[35] Andre, P. Globally representative evidence on the actual and perceived support for climate action. *Nat. Clim. Chang* **14**, 253–259 (2024).

[36] Kasperson, R. E. et al. The social amplification of risk: a conceptual framework. *Risk Anal.* **8**, 177–187 (1988).

[37] Kasperson, R. E., Webler, T., Ram, B. & Sutton, J. The social amplification of risk framework: new perspectives. *Risk Anal.* **42**, 1367–1380 (2022). 35861634 10.1111/risa.13926 PMC10360138

[38] Zhang, Y., Bai, X., Mills, F. P. & Pezzey, J. C. V. Examining the attitude-behavior gap in residential energy use: empirical evidence from a large-scale survey in Beijing, China. *J. Clean. Prod.* **295**, 126510 (2021).

[39] Nielsen, K. S. et al. The motivation–impact gap in pro-environmental clothing consumption. *Nat. Sustain.* **5**, 665–668 (2022).

[40] The attitude-behavior gap on climate action: how can it be bridged? *Yale Program on Climate Change Communication* https://climatecommunication.yale.edu/publications/attitude-behavior-gap/.

[41] Vieira, J., Castro, S. L. & Souza, A. S. Psychological barriers moderate the attitude-behavior gap for climate change. *PLoS ONE* **18**, e0287404 (2023). 37405976 10.1371/journal.pone.0287404 PMC10321650

[42] Nielsen, K. S. et al. Perceived plasticity of climate-relevant behaviors and policy support among high- and lower-income individuals. Preprint at https://osf.io/x6473.

[43] Donelson, J. M. et al. Putting plasticity into practice for effective conservation actions under climate change. *Nat. Clim. Chang.* **13**, 632–647 (2023).

[44] Boudet, H., Giordono, L., Zanocco, C., Satein, H. & Whitley, H. Event attribution and partisanship shape local discussion of climate change after extreme weather. *Nat. Clim. Chang.* **10**, 69–76 (2020).

[45] Caggiano, H., Constantino, S. M., Greig, C. & Weber, E. U. Public and local policymaker preferences for large-scale energy project characteristics. *Nat. Energy* 1–11. 10.1038/s41560-024-01603-w (2024).

[46] Ogunbode, C. A., Doran, R. & Böhm, G. Individual and local flooding experiences are differentially associated with subjective attribution and climate change concern. *Clim. Chang.* **162**, 2243–2255 (2020).

[47] Laurice Jamero, M. A. et al. Small-island communities in the Philippines prefer local measures to relocation in response to sea-level rise. *Nat. Clim. Chang.* **7**, 581–586 (2017).

[48] Tomczak, M. Spatial interpolation and its uncertainty using automated anisotropic inverse distance weighting (IDW)-cross-validation/jackknife approach. *J. Geogr. Inf. Decis. Anal.* **2**, 18–30 (1998).

[49] Buttice, M. K. & Highton, B. How does multilevel regression and poststratification perform with conventional national surveys? *Polit. Anal.* **21**, 449–467 (2013).

[50] Broniecki, P., Leemann, L. & Wüest, R. Improved multilevel regression with poststratification through machine learning (autoMrP). *J. Polit.* **84**, 597–601 (2022).

[51] Downes, M. et al. Multilevel regression and poststratification: a modeling approach to estimating population quantities from highly selected survey samples. *Am. J. Epidemiol.* **187**, 1780–1790 (2018). 29635276 10.1093/aje/kwy070

[52] Zhang, X. et al. Validation of multilevel regression and poststratification methodology for small area estimation of health indicators from the behavioral risk factor surveillance system. *Am. J. Epidemiol.* **182**, 127–137 (2015). 25957312 10.1093/aje/kwv002 PMC4554328

[53] Hanretty, C. An introduction to multilevel regression and post-stratification for estimating constituency opinion. *Polit. Stud. Rev.* **18**, 630–645 (2020).

[54] Ghitza, Y. & Gelman, A. Voter registration databases and MRP: toward the use of large-scale databases in public opinion research. *Polit. Anal.* **28**, 507–531 (2020).

[55] Lee, T. M., Markowitz, E. M., Howe, P. D., Ko, C.-Y. & Leiserowitz, A. A. Predictors of public climate change awareness and risk perception around the world. *Nat. Clim. Chang.* **5**, 1014–1020 (2015).

[56] Clarke, B., Otto, F., Stuart-Smith, R. & Harrington, L. Extreme weather impacts of climate change: an attribution perspective. *Environ. Res. Clim.* **1**, 012001 (2022).

[57] McCright, A. M., Dunlap, R. E. & Xiao, C. The impacts of temperature anomalies and political orientation on perceived winter warming. *Nat. Clim. Chang.* **4**, 1077–1081 (2014).

[58] Sugerman, E. R., Li, Y. & Johnson, E. J. Local warming is real: a meta-analysis of the effect of recent temperature on climate change beliefs. *Curr. Opin. Behav. Sci.* **42**, 121–126 (2021).

[59] Marlon, J. R. et al. Hot dry days increase perceived experience with global warming. *Glob. Environ. Change* **68**, 102247 (2021).

[60] Anselin, L. Spatial econometrics: methods and models. Vol. 4. (Springer Science & Business Media, 1988).

[61] Anselin, L., Syabri, I. & Kho, Y. GeoDa: an introduction to spatial data analysis. Handbook of applied spatial analysis: Software tools, methods and applications. 73−89 (Berlin, Heidelberg: Springer Berlin Heidelberg, 2009).

[62] Getis, A. Spatial autocorrelation. in *Handbook of Applied Spatial Analysis: Software Tools, Methods and Applications* 255–278 (Springer, 2009).

[63] Blozis, S. A., McTernan, M., Harring, J. R. & Zheng, Q. Two-part mixed-effects location scale models. *Behav. Res. Methods* **52**, 1836–1847 (2020). 32043225 10.3758/s13428-020-01359-7

[64] Brunton-Smith, I., Sturgis, P. & Leckie, G. Detecting and understanding interviewer effects on survey data by using a cross-classified mixed effects location–scale model. *J. R. Stat. Soc. Ser. A Stat. Soc*. **180**, 551–568 (2017).

[65] Arias, P. A. et al. Technical Summary. In Climate Change 2021: The Physical Science Basis. Contribution of Working Group I to the Sixth Assessment Report of the Intergovernmental Panel on Climate Change (eds Masson-Delmotte, V. P., et al.) 33−144. (Cambridge University Press, Cambridge, United Kingdom and New York, NY, USA 2021).

[66] Speizer, S., Raymond, C., Ivanovich, C. & Horton, R. M. Concentrated and intensifying humid heat extremes in the IPCC AR6 regions. *Geophys. Res. Lett.* **49**, e2021GL097261 (2022).

