# Supplementary material for: Growing climate change risk concerns with rising regional disparities in China
Source: NPJ Clim Action. 2025 Aug 20;4(1):78. doi: 10.1038/s44168-025-00272-z (PMC12367554; doi:10.1038/s44168-025-00272-z)
Supplement: Supplementary file 1 — Supplementary materials-SUBMIT [file 44168_2025_272_MOESM1_ESM.pdf]

Supplementary materials for

Growing climate change concern but rising regional disparities in China

Table S1 Demographic characteristics of respondents. Error! Bookmark not defined.

Table S2 Operationalization of causal conditions. 4

Fig. S1 Correlation between Perceived Priority and Perceived Impact of Climate Change.. 6

Fig. S2 Influencing Factors of Climate Change Perception in China.. 7

Fig. S3 Mean Absolute Error (MAE) comparison between Multilevel Regression and Poststratification (MRP) and Disaggregation methods over repeated simulations. 8

Fig. S4 Comparison of actual versus perceived impact rankings of climate change in China for 2010 and 2023....9

Table S1 Demographic characteristics of respondents. Comparison of the demographic characteristics between the participants of our survey conducted in 2022 and those from the China General Social Survey (CGSS) conducted in 2010. Percentages may not sum to 100% due to rounding. Respondents are categorised into groups based on gender, age, education, family income (USD per month), residence, and occupation. Occupation groupings for the CGSS survey are not reported.

Factors	Groups	Our survey (2022)		CGSS (2010)	
		Sample size	Percentage (%)	Sample size	Percentage (%)
Gender	Male	1,387	34.2	5677	48.2
	Female	2,663	65.8	6106	51.8
Age	<25	1,438	35.5	906	7.69
	26~35	1,156	28.5	2210	18.8
	35~45	796	19.7	2514	21.3
	>46	660	15.6	6150	52.2
Education	High school or lower	3,139	77.5	9350	91.4
	College or higher	911	22.5	880	8.60
Family income (USD/month)	<800	654	16.1	964	9.33
	800~1,500	1,356	33.5	1213	11.7
	1,500~3,000	1,313	32.4	2383	23.1
	>3,000	727	18.0	5774	55.9
Residence	Urban	3,113	76.9	7222	61.3
	Rural	937	23.1	4561	38.7
Occupation	Student	1,052	26.0		
	Civil servant	365	9.0		
	Resource or energy-related	476	11.8		
	Agriculture-related	234	5.8		
	Outdoor workers	218	5.4		
	Others	1,705	42.0		

Table S2 Operationalization of causal conditions.

Condition	Scoring		External Source
Extreme Environmental Events	0-1	Thresholds for extreme events were determined for each city based on the 98th percentile of historical daily average temperatures from 1980 through 2020	
Media Exposure	0-1	Baidu Search Index for Climate Change	15–17
Economic Prosperity	0-1	GDP per Capita	
Educational Levels	0-1	Number of students enrolled in higher education in each province.	
Baseline Perception	0-1	Determined based on the recorded perception in 2010	

Table S3 Analysis of conditions associated with increases in perceived priority and impact of climate change. The table presents metrics for both necessity and sufficiency of each condition. Inclusion scores reflect the extent to which a condition is present in all instances of the outcome, with values closer to 1 indicating higher necessity. The Ratio of Necessity and the Ratio of Sufficiency denote the proportion of cases where the condition is necessary or sufficient, respectively, for the outcome. Coverage scores measure the proportion of the outcome explained by the condition, with higher values indicating greater explanatory power.

**Condition**	**Inclusion (Necessary)**	**Ratio of Necessity**	**Coverage (Necessary)**	**Inclusion (Sufficient)**	**Ratio of Sufficiency**	**Coverage (Sufficient)**
**Increasing in Perceived Priority**						
Baseline Perceived Priority	0.501	0.862	0.851	0.704	0.527	0.165
Extreme Weather Events	0.599	0.685	0.572	0.569	0.677	0.556
Media Exposure	0.637	0.714	0.627	0.506	0.641	0.479
GDP per Capita	0.565	0.661	0.523	0.566	0.684	0.569
Education Levels	0.675	0.696	0.594	0.473	0.649	0.497
**Increasing in Perceived Impact**						
Baseline Perceived Impact	0.545	1	1	0.001	0.5	0
Extreme Weather Events	0.489	0.659	0.483	0.622	0.73	0.63
Internet Searches	0.503	0.672	0.513	0.597	0.707	0.585
GDP per Capita	0.582	0.693	0.558	0.567	0.709	0.59
Education Levels	0.514	0.653	0.469	0.58	0.73	0.631

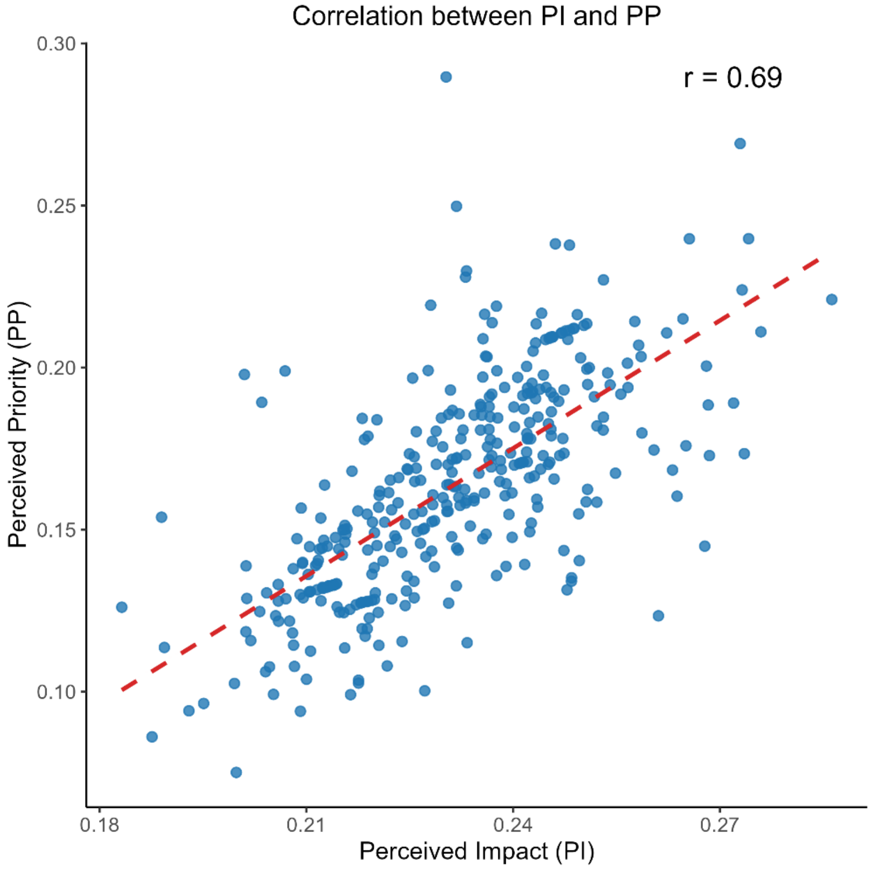

Fig.S1 Correlation between Perceived Priority and Perceived Impact of Climate Change. The scatter plot depicts the strong positive relationship between Perceived Priority and Perceived Impact across MRP estimation, demonstrating a correlation coefficient of 0.69. Each point represents an individual city, with their Perceived Priority and Perceived Impact scores plotted on the x and y axes, respectively.

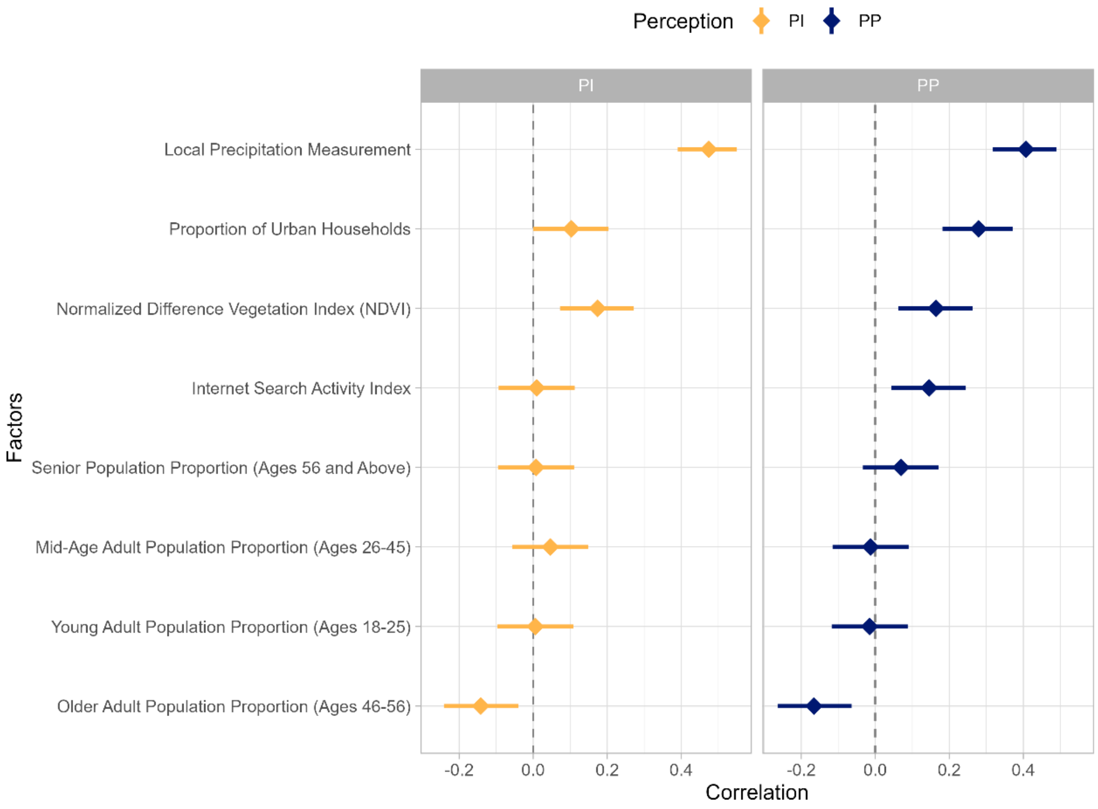

Fig. S2 Influencing Factors of Climate Change Perception in China. The forest plot displays the city-level scale-down estimates of Multilevel Regression with Poststratification (MRP) against a range of demographic, environmental, and societal factors impacting climate change perception across China. Each influencing factor is represented by a diamond marker indicating the estimate, with horizontal lines illustrating the confidence interval.

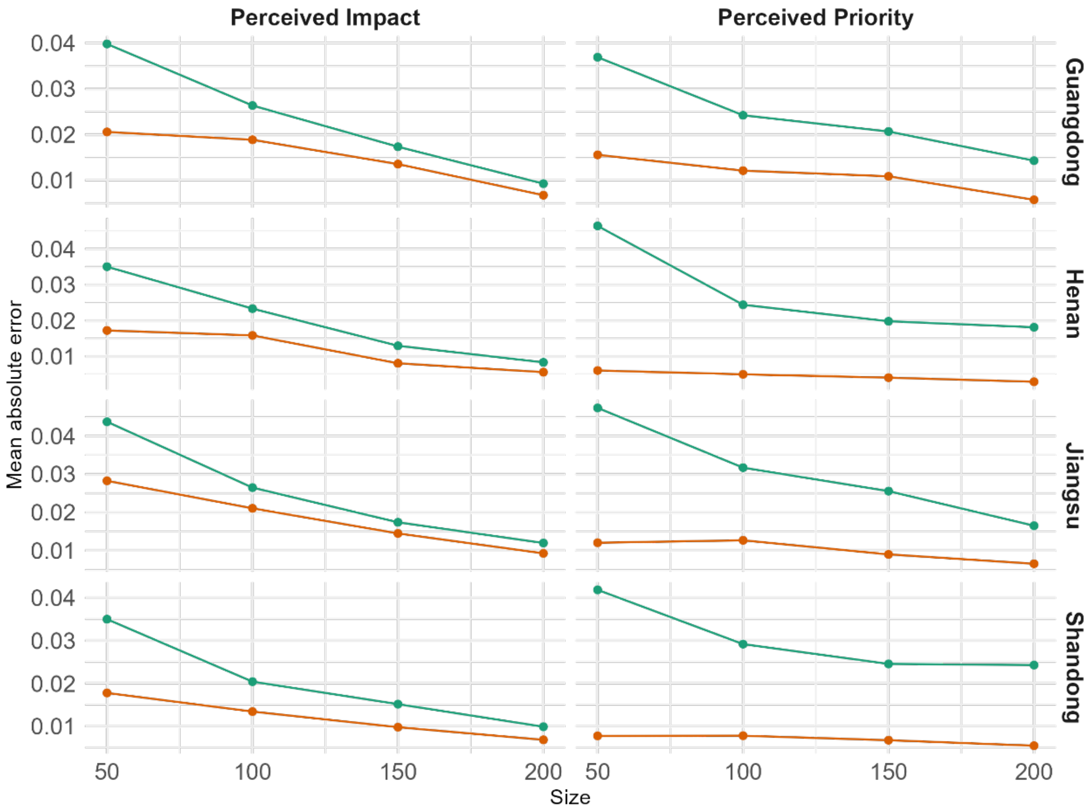

Fig. S3 Mean Absolute Error (MAE) comparison between Multilevel Regression and Poststratification (MRP) and Disaggregation methods over repeated simulations. The figure illustrates the MAE for Perceived Priority (PP) and Perceived Impact (PI) of climate change, derived from two methods - MRP and Disaggregation - across different sample sizes and provinces. The sample sizes are determined based on 99 random samplings of 50, 100, 150, and 200 responses from the survey data. The different panels correspond to different provinces (shown on the vertical axis). This comparison validates the robustness of MRP estimates across varying sample sizes, demonstrating its effectiveness in estimating climate change perceptions, even with small sample sizes.

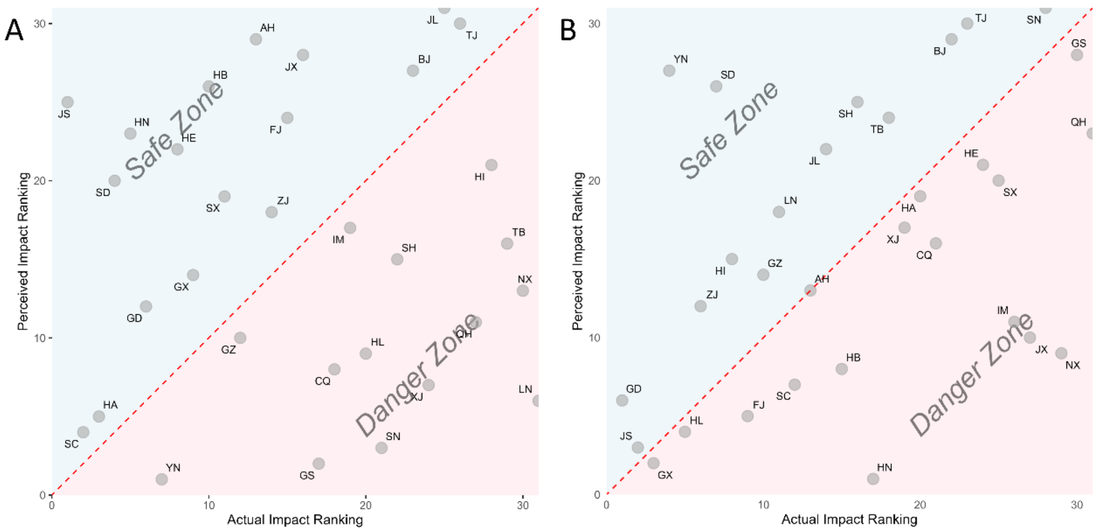

Fig. S4 Comparison of actual versus perceived impact rankings of climate change in China for 2010 and 2023. A) The 2010 data illustrate the initial relationship between perceived and actual impacts of climate change across various provinces, with a dotted red line indicating parity between the two measures. Provinces above the line are in the 'Safe Zone', where perception exceeds actual risk, while those below the line fall into the 'Danger Zone', indicating underestimation of risk. B) The 2023 data display the evolved perceptions, showing shifts in provinces' positions relative to the parity line. The distribution of provinces across the 'Safe Zone' and 'Danger Zone' reflects changes over the 13-year period in the public's alignment of perceived impact with actual climate risk.
